# [Bis(3,5-dimethyl­pyrazol-1-yl)methane]{*N*-[1-(2-oxidophen­yl)ethyl­idene]-dl-alaninato}copper(II) monohydrate

**DOI:** 10.1107/S1600536808037264

**Published:** 2008-11-13

**Authors:** Gan-Qing Zhao, Ling-Wei Xue, Cheng-Jun Hao, Li-Hua Chen, Hua-Tao Wu

**Affiliations:** a School of Chemistry and Chemical Engineering, Pingdingshan University, Pingdingshan 467000, People’s Republic of China

## Abstract

In the title compound, [Cu(C_11_H_11_NO_3_)(C_11_H_16_N_4_)]·H_2_O, the Cu^II^ atom is five-coordinate in a distorted square-pyramidal geometry. The basal positions are occupied by three donor atoms from the tridentate Schiff base ligand and by one N atom from a bis­(3,5-dimethyl­prazol-l-yl)methane ligand. The apical position is occupied by the N atom of the other ligand of this type. There are only van der Waals contacts in the crystal structure.

## Related literature

For background to transition metal complexes with Schiff base ligands, see: Casella & Guillotti (1983 ▶); Ganguly *et al.* (2008 ▶); Vigato & Tamburini (2004 ▶). For structural studies of Schiff base complexes derived from 2-hydroxy­acetophenone and animo acids, see: Baul *et al.* (2007 ▶); Parekh *et al.* (2006 ▶); Usman *et al.* (2003 ▶). For related literature, see: Plesch *et al.* (1997 ▶).
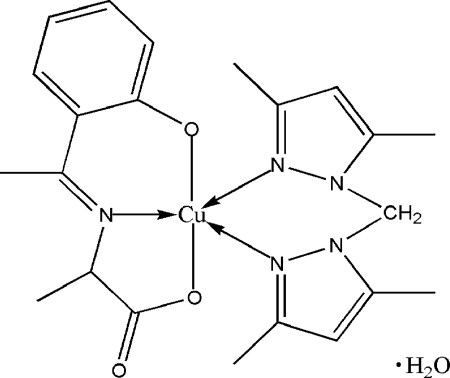


## Experimental

### 

#### Crystal data

[Cu(C_11_H_11_NO_3_)(C_11_H_16_N_4_)]·H_2_O
*M* *_r_* = 491.04
Monoclinic, 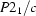

*a* = 13.365 (3) Å
*b* = 7.8602 (15) Å
*c* = 23.404 (4) Å
β = 102.315 (2)°
*V* = 2402.1 (8) Å^3^
*Z* = 4
Mo *K*α radiation
μ = 0.95 mm^−1^
*T* = 293 (2) K
0.36 × 0.25 × 0.20 mm

#### Data collection

Bruker SMART CCD area-detector diffractometer
Absorption correction: multi-scan (*SADABS*; Sheldrick, 1996 ▶) *T*_min_ = 0.727, *T*_max_ = 0.833
14432 measured reflections
5500 independent reflections
3724 reflections with *I* > 2σ(*I*)
*R*_int_ = 0.054

#### Refinement

*R*[*F* ^2^ > 2σ(*F* ^2^)] = 0.052
*wR*(*F* ^2^) = 0.150
*S* = 1.02
5500 reflections
296 parameters
H-atom parameters constrained
Δρ_max_ = 0.79 e Å^−3^
Δρ_min_ = −0.53 e Å^−3^

### 

Data collection: *SMART* (Bruker, 2000 ▶); cell refinement: *SAINT* (Bruker, 2000 ▶); data reduction: *SAINT*; program(s) used to solve structure: *SHELXS97* (Sheldrick, 2008 ▶); program(s) used to refine structure: *SHELXL97* (Sheldrick, 2008 ▶); molecular graphics: *SHELXTL* (Sheldrick, 2008 ▶); software used to prepare material for publication: *SHELXTL*.

## Supplementary Material

Crystal structure: contains datablocks global, I. DOI: 10.1107/S1600536808037264/bq2096sup1.cif

Structure factors: contains datablocks I. DOI: 10.1107/S1600536808037264/bq2096Isup2.hkl

Additional supplementary materials:  crystallographic information; 3D view; checkCIF report

## Figures and Tables

**Table 1:** Selected bond lengths (Å)

Cu1—O1	1.879 (2)
Cu1—O2	1.961 (2)
Cu1—N1	1.974 (2)
Cu1—N4	2.062 (2)
Cu1—N2	2.315 (3)

**Fig. 1.**
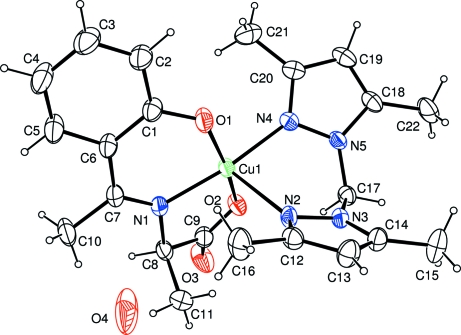
The structure of the title compound, showing 30% probability displacement ellipsoids and the atom-numbering scheme.

## supplementary crystallographic information

### Comment

In the past decades, significant progress has been achieved in understanding the chemistry of transition metal complexes with Schiff base ligands composed of salicylaldehyde, 2-formylpyridine or their analogues, and α-amino acids (Vigato & Tamburini, 2004; Ganguly *et al.*, 2008; Casella & Guillotti, 1983). A few stuctural studies have been performed on Schiff base complexes derived from 2-Hydroxyacetophenone and animo acids (Usman *et al.*, 2003; Baul *et al.*, 2007; Parekh *et al.*, 2006). We report here the crystal structure of the title Cu^II^ complex, (I).

The structure consists of discrete monomeric square-pyramidal Cu^II^ complex (Fig. 1 and Table 1). The basal positions are occupied by three donor atoms from the tridentate Schiff base ligand, which furnishes an ONO donor set, with the fourth position occupied by one N atom from the 1,1-bis(3,5-dimethylprazol-l-yl)methane ligand. The apical position is occupied by the other N atom of this ligand.

The two nitrogen heterocycles are planar and lie at angles of 95.5° and 30.9° to the plane of the C1—C6 ring. The two nitrogen heterocycles form a dihedral angle of 66.2° with each other.

The van der Waals contacts are major factors in the crystal packing. The H atoms of water could not be fixed because of the high disorder of O4. So, no comment can be given about the probable O—H···O type hydrogen bonds which should be formed through the solvent water molecule with neighboring carboxylate oxygen O3.

### Experimental

The title compound was synthesized as described in the literature (Plesch *et al.*, 1997). To *L*-valine (1.00 mmol) and potassium hydroxide (1.00 mmol) in 10 ml of methanol was added 2-Hydroxyacetophenone (1.00 mmol in 10 ml of methanol) dropwise. The yellow solution was stirred for 2.0 h at 333 K. The resultant mixture was added dropwise to copper (II) acetate monohydrate (1.00 mmol) and 1,1-bis(3,5-dimethylprazol-l-yl)methane (1.00 mmol) in an aqueous methanolic solution (20 ml, 1:1 *v*/*v*), and heated with stirring for 2.0 h at 333 K. The dark blue solution was filtered and left for several days, dark blue crystals had formed that were filtered off, washed with water, and dried under vacuum.

### Refinement

In (I), All H atoms were positioned geometrically and refined as riding atoms, with C—H = 0.93 (CH) or 0.97 Å (CH_2_) and *U*_iso_(H) = 1.2*U*_eq_(C), with C—H = 0.96 Å (CH_3_) and *U*_iso_(H) = 1.5*U*_eq_(C). The oxygen (O4) of the water molecule is extremely disorder. So, no H-atom could be attached.

### Crystal data

**Table:**

[Cu(C_11_H_11_NO_3_)(C_11_H_16_N_4_)]·H_2_O_1_	*F*_000_ = 1028
*M**_r_* = 491.04	*D*_x_ = 1.358 Mg m^−^^3^
Monoclinic, *P*2_1_/*c*	Mo *K*α radiation λ = 0.71073 Å
Hall symbol: -P 2ybc	Cell parameters from 3344 reflections
*a* = 13.365 (3) Å	θ = 2.6–23.9º
*b* = 7.8602 (15) Å	µ = 0.95 mm^−^^1^
*c* = 23.404 (4) Å	*T* = 293 (2) K
β = 102.315 (2)º	Block, dark blue
*V* = 2402.1 (8) Å^3^	0.36 × 0.25 × 0.20 mm
*Z* = 4	

### Data collection

**Table:**

Bruker SMART CCD area-detector diffractometer	5500 independent reflections
Radiation source: fine-focus sealed tube	3724 reflections with *I* > 2σ(*I*)
Monochromator: graphite	*R*_int_ = 0.054
*T* = 293(2) K	θ_max_ = 27.6º
φ and ω scans	θ_min_ = 2.1º
Absorption correction: multi-scan(SADABS; Sheldrick, 1996)	*h* = −12→17
*T*_min_ = 0.727, *T*_max_ = 0.833	*k* = −10→10
14432 measured reflections	*l* = −30→27

### Refinement

**Table:**

Refinement on *F*^2^	Secondary atom site location: difference Fourier map
Least-squares matrix: full	Hydrogen site location: inferred from neighbouring sites
*R*[*F*^2^ > 2σ(*F*^2^)] = 0.052	H-atom parameters constrained
*wR*(*F*^2^) = 0.150	*w* = 1/[σ^2^(*F*_o_^2^) + (0.0715*P*)^2^ + 0.4661*P*] where *P* = (*F*_o_^2^ + 2*F*_c_^2^)/3
*S* = 1.02	(Δ/σ)_max_ = 0.001
5500 reflections	Δρ_max_ = 0.79 e Å^−^^3^
296 parameters	Δρ_min_ = −0.53 e Å^−^^3^
Primary atom site location: structure-invariant direct methods	Extinction correction: none

### Special details

**Table:**

Geometry. All e.s.d.'s (except the e.s.d. in the dihedral angle between two l.s. planes) are estimated using the full covariance matrix. The cell e.s.d.'s are taken into account individually in the estimation of e.s.d.'s in distances, angles and torsion angles; correlations between e.s.d.'s in cell parameters are only used when they are defined by crystal symmetry. An approximate (isotropic) treatment of cell e.s.d.'s is used for estimating e.s.d.'s involving l.s. planes.
Refinement. Refinement of *F*^2^ against ALL reflections. The weighted *R*-factor *wR* and goodness of fit *S* are based on *F*^2^, conventional *R*-factors *R* are based on *F*, with *F* set to zero for negative *F*^2^. The threshold expression of *F*^2^ > σ(*F*^2^) is used only for calculating *R*-factors(gt) *etc*. and is not relevant to the choice of reflections for refinement. *R*-factors based on *F*^2^ are statistically about twice as large as those based on *F*, and *R*- factors based on ALL data will be even larger.

### Fractional atomic coordinates and isotropic or equivalent isotropic displacement parameters (Å^2^)

**Table:**

	*x*	*y*	*z*	*U*_iso_*/*U*_eq_	
C1	0.8201 (2)	0.6577 (4)	0.77226 (13)	0.0495 (8)	
C2	0.8236 (3)	0.6612 (6)	0.83301 (14)	0.0657 (10)	
H2	0.7623	0.6597	0.8457	0.079*	
C3	0.9132 (3)	0.6666 (6)	0.87380 (16)	0.0758 (12)	
H3	0.9122	0.6682	0.9134	0.091*	
C4	1.0060 (3)	0.6697 (6)	0.85614 (16)	0.0777 (12)	
H4	1.0675	0.6774	0.8835	0.093*	
C5	1.0049 (3)	0.6611 (5)	0.79729 (16)	0.0657 (10)	
H5	1.0673	0.6589	0.7858	0.079*	
C6	0.9140 (2)	0.6554 (4)	0.75344 (14)	0.0473 (8)	
C7	0.9202 (2)	0.6387 (4)	0.69200 (14)	0.0460 (7)	
C8	0.8453 (2)	0.5740 (4)	0.59087 (13)	0.0477 (8)	
H8	0.9104	0.5182	0.5896	0.057*	
C9	0.7567 (3)	0.4588 (5)	0.56306 (14)	0.0519 (8)	
C10	1.0249 (2)	0.6546 (6)	0.67706 (17)	0.0690 (11)	
H10A	1.0172	0.6609	0.6354	0.104*	
H10B	1.0657	0.5571	0.6917	0.104*	
H10C	1.0581	0.7558	0.6947	0.104*	
C11	0.8367 (3)	0.7413 (5)	0.55651 (16)	0.0692 (11)	
H11A	0.8971	0.8084	0.5701	0.104*	
H11B	0.7777	0.8032	0.5622	0.104*	
H11C	0.8300	0.7171	0.5157	0.104*	
C12	0.6251 (2)	0.9931 (4)	0.62768 (14)	0.0477 (7)	
C13	0.5374 (3)	1.0828 (4)	0.60353 (17)	0.0594 (9)	
H13	0.5290	1.2003	0.6039	0.071*	
C14	0.4655 (3)	0.9659 (4)	0.57912 (15)	0.0510 (8)	
C15	0.3571 (3)	0.9869 (6)	0.5464 (2)	0.0819 (13)	
H15A	0.3469	0.9248	0.5103	0.123*	
H15B	0.3432	1.1053	0.5383	0.123*	
H15C	0.3115	0.9440	0.5697	0.123*	
C16	0.7269 (3)	1.0596 (5)	0.66044 (19)	0.0730 (12)	
H16A	0.7522	0.9881	0.6936	0.110*	
H16B	0.7186	1.1735	0.6735	0.110*	
H16C	0.7747	1.0595	0.6351	0.110*	
C17	0.4717 (2)	0.6429 (4)	0.57657 (12)	0.0411 (7)	
H17A	0.5156	0.5804	0.5559	0.049*	
H17B	0.4041	0.6494	0.5513	0.049*	
C18	0.3806 (2)	0.5024 (5)	0.64872 (15)	0.0504 (8)	
C19	0.4159 (3)	0.4218 (4)	0.70075 (16)	0.0586 (9)	
H19	0.3761	0.3709	0.7241	0.070*	
C20	0.5217 (3)	0.4297 (4)	0.71236 (14)	0.0491 (8)	
C21	0.5955 (3)	0.3618 (6)	0.76487 (17)	0.0787 (12)	
H21A	0.6578	0.3282	0.7538	0.118*	
H21B	0.5657	0.2651	0.7801	0.118*	
H21C	0.6101	0.4486	0.7943	0.118*	
C22	0.2745 (3)	0.5421 (6)	0.61745 (19)	0.0811 (13)	
H22A	0.2645	0.5038	0.5777	0.122*	
H22B	0.2635	0.6627	0.6180	0.122*	
H22C	0.2268	0.4854	0.6364	0.122*	
Cu1	0.69565 (3)	0.58580 (5)	0.659531 (15)	0.03989 (15)	
N1	0.83952 (18)	0.6059 (3)	0.65209 (11)	0.0421 (6)	
N2	0.61092 (17)	0.8286 (3)	0.61916 (11)	0.0432 (6)	
N3	0.51185 (17)	0.8133 (3)	0.58899 (10)	0.0402 (6)	
N4	0.55252 (18)	0.5077 (3)	0.66850 (11)	0.0428 (6)	
N5	0.46534 (18)	0.5521 (3)	0.62967 (11)	0.0411 (6)	
O1	0.72874 (16)	0.6610 (3)	0.73752 (9)	0.0561 (6)	
O2	0.67815 (16)	0.4594 (3)	0.58584 (9)	0.0503 (6)	
O3	0.7635 (2)	0.3745 (4)	0.51962 (12)	0.0863 (10)	
O4	0.9427 (3)	0.3264 (13)	0.48232 (19)	0.269 (5)	

### Atomic displacement parameters (Å^2^)

**Table:**

	*U*^11^	*U*^22^	*U*^33^	*U*^12^	*U*^13^	*U*^23^
C1	0.0451 (18)	0.054 (2)	0.0463 (18)	0.0114 (15)	0.0040 (15)	−0.0053 (15)
C2	0.057 (2)	0.096 (3)	0.0428 (19)	0.016 (2)	0.0085 (17)	−0.0079 (18)
C3	0.077 (3)	0.102 (3)	0.043 (2)	0.019 (2)	0.003 (2)	−0.007 (2)
C4	0.059 (3)	0.110 (4)	0.053 (2)	0.023 (2)	−0.0117 (19)	−0.013 (2)
C5	0.0406 (19)	0.087 (3)	0.064 (2)	0.0130 (19)	−0.0011 (17)	−0.010 (2)
C6	0.0365 (17)	0.0529 (19)	0.0495 (18)	0.0100 (14)	0.0022 (14)	−0.0056 (15)
C7	0.0319 (16)	0.0505 (19)	0.0542 (19)	0.0051 (13)	0.0063 (14)	0.0006 (15)
C8	0.0319 (16)	0.071 (2)	0.0423 (17)	0.0032 (15)	0.0116 (13)	−0.0015 (15)
C9	0.0422 (18)	0.072 (2)	0.0412 (17)	0.0053 (16)	0.0081 (14)	−0.0056 (15)
C10	0.0305 (18)	0.105 (3)	0.071 (2)	−0.0030 (19)	0.0106 (17)	−0.013 (2)
C11	0.058 (2)	0.093 (3)	0.061 (2)	−0.001 (2)	0.0220 (18)	0.019 (2)
C12	0.0421 (18)	0.0425 (19)	0.058 (2)	−0.0077 (14)	0.0094 (15)	0.0009 (15)
C13	0.057 (2)	0.0430 (19)	0.072 (2)	0.0043 (16)	0.0015 (19)	−0.0004 (16)
C14	0.0444 (18)	0.052 (2)	0.055 (2)	0.0094 (15)	0.0066 (15)	0.0041 (15)
C15	0.056 (2)	0.079 (3)	0.096 (3)	0.021 (2)	−0.015 (2)	−0.001 (3)
C16	0.051 (2)	0.064 (3)	0.096 (3)	−0.0145 (18)	−0.002 (2)	−0.009 (2)
C17	0.0341 (16)	0.0482 (18)	0.0402 (16)	−0.0048 (13)	0.0059 (13)	−0.0052 (13)
C18	0.0394 (17)	0.059 (2)	0.057 (2)	−0.0143 (16)	0.0188 (15)	−0.0086 (16)
C19	0.054 (2)	0.068 (2)	0.062 (2)	−0.0176 (17)	0.0295 (18)	−0.0038 (18)
C20	0.058 (2)	0.0458 (19)	0.0480 (18)	−0.0049 (15)	0.0206 (16)	0.0004 (14)
C21	0.088 (3)	0.084 (3)	0.063 (2)	−0.001 (2)	0.015 (2)	0.027 (2)
C22	0.037 (2)	0.121 (4)	0.087 (3)	−0.013 (2)	0.019 (2)	0.006 (3)
Cu1	0.0293 (2)	0.0490 (3)	0.0421 (2)	0.00231 (15)	0.00949 (15)	−0.00283 (16)
N1	0.0319 (13)	0.0521 (16)	0.0421 (14)	0.0036 (11)	0.0074 (11)	0.0015 (11)
N2	0.0263 (12)	0.0471 (16)	0.0528 (15)	−0.0040 (11)	0.0008 (11)	0.0023 (12)
N3	0.0311 (13)	0.0443 (15)	0.0441 (13)	0.0003 (11)	0.0056 (11)	0.0014 (11)
N4	0.0390 (14)	0.0445 (15)	0.0462 (14)	−0.0040 (12)	0.0119 (12)	0.0031 (12)
N5	0.0309 (13)	0.0526 (16)	0.0414 (14)	−0.0067 (11)	0.0111 (11)	−0.0009 (11)
O1	0.0336 (12)	0.0861 (17)	0.0478 (13)	0.0080 (11)	0.0069 (10)	−0.0120 (12)
O2	0.0379 (12)	0.0612 (15)	0.0548 (13)	−0.0025 (10)	0.0167 (10)	−0.0111 (10)
O3	0.0584 (17)	0.142 (3)	0.0650 (17)	−0.0115 (16)	0.0265 (14)	−0.0473 (17)
O4	0.082 (3)	0.624 (14)	0.103 (3)	0.110 (5)	0.026 (2)	−0.007 (5)

### Geometric parameters (Å, °)

**Table:**

C1—O1	1.314 (4)	C14—C15	1.497 (4)
C1—C2	1.413 (4)	C15—H15A	0.9600
C1—C6	1.417 (4)	C15—H15B	0.9600
C2—C3	1.364 (5)	C15—H15C	0.9600
C2—H2	0.9300	C16—H16A	0.9600
C3—C4	1.388 (6)	C16—H16B	0.9600
C3—H3	0.9300	C16—H16C	0.9600
C4—C5	1.376 (5)	C17—N3	1.449 (4)
C4—H4	0.9300	C17—N5	1.451 (4)
C5—C6	1.414 (4)	C17—H17A	0.9700
C5—H5	0.9300	C17—H17B	0.9700
C6—C7	1.464 (4)	C18—N5	1.361 (4)
C7—N1	1.293 (4)	C18—C19	1.364 (5)
C7—C10	1.518 (4)	C18—C22	1.483 (5)
C8—N1	1.473 (4)	C19—C20	1.384 (5)
C8—C9	1.522 (5)	C19—H19	0.9300
C8—C11	1.533 (5)	C20—N4	1.334 (4)
C8—H8	0.9800	C20—C21	1.500 (5)
C9—O3	1.233 (4)	C21—H21A	0.9600
C9—O2	1.275 (4)	C21—H21B	0.9600
C10—H10A	0.9600	C21—H21C	0.9600
C10—H10B	0.9600	C22—H22A	0.9600
C10—H10C	0.9600	C22—H22B	0.9600
C11—H11A	0.9600	C22—H22C	0.9600
C11—H11B	0.9600	Cu1—O1	1.879 (2)
C11—H11C	0.9600	Cu1—O2	1.961 (2)
C12—N2	1.316 (4)	Cu1—N1	1.974 (2)
C12—C13	1.381 (5)	Cu1—N4	2.062 (2)
C12—C16	1.505 (5)	Cu1—N2	2.315 (3)
C13—C14	1.364 (5)	N2—N3	1.367 (3)
C13—H13	0.9300	N4—N5	1.362 (3)
C14—N3	1.348 (4)		
			
O1—C1—C2	116.7 (3)	H16A—C16—H16B	109.5
O1—C1—C6	125.1 (3)	C12—C16—H16C	109.5
C2—C1—C6	118.1 (3)	H16A—C16—H16C	109.5
C3—C2—C1	122.8 (4)	H16B—C16—H16C	109.5
C3—C2—H2	118.6	N3—C17—N5	111.7 (2)
C1—C2—H2	118.6	N3—C17—H17A	109.3
C2—C3—C4	119.9 (3)	N5—C17—H17A	109.3
C2—C3—H3	120.0	N3—C17—H17B	109.3
C4—C3—H3	120.0	N5—C17—H17B	109.3
C5—C4—C3	118.6 (3)	H17A—C17—H17B	107.9
C5—C4—H4	120.7	N5—C18—C19	105.8 (3)
C3—C4—H4	120.7	N5—C18—C22	123.6 (3)
C4—C5—C6	123.5 (4)	C19—C18—C22	130.6 (3)
C4—C5—H5	118.3	C18—C19—C20	107.4 (3)
C6—C5—H5	118.3	C18—C19—H19	126.3
C5—C6—C1	117.1 (3)	C20—C19—H19	126.3
C5—C6—C7	119.7 (3)	N4—C20—C19	109.9 (3)
C1—C6—C7	123.1 (3)	N4—C20—C21	122.5 (3)
N1—C7—C6	120.9 (3)	C19—C20—C21	127.6 (3)
N1—C7—C10	121.2 (3)	C20—C21—H21A	109.5
C6—C7—C10	117.8 (3)	C20—C21—H21B	109.5
N1—C8—C9	108.6 (2)	H21A—C21—H21B	109.5
N1—C8—C11	110.5 (3)	C20—C21—H21C	109.5
C9—C8—C11	108.8 (3)	H21A—C21—H21C	109.5
N1—C8—H8	109.6	H21B—C21—H21C	109.5
C9—C8—H8	109.6	C18—C22—H22A	109.5
C11—C8—H8	109.6	C18—C22—H22B	109.5
O3—C9—O2	124.0 (3)	H22A—C22—H22B	109.5
O3—C9—C8	119.0 (3)	C18—C22—H22C	109.5
O2—C9—C8	117.0 (3)	H22A—C22—H22C	109.5
C7—C10—H10A	109.5	H22B—C22—H22C	109.5
C7—C10—H10B	109.5	O1—Cu1—O2	166.62 (10)
H10A—C10—H10B	109.5	O1—Cu1—N1	91.65 (10)
C7—C10—H10C	109.5	O2—Cu1—N1	84.17 (9)
H10A—C10—H10C	109.5	O1—Cu1—N4	91.49 (10)
H10B—C10—H10C	109.5	O2—Cu1—N4	89.98 (9)
C8—C11—H11A	109.5	N1—Cu1—N4	167.22 (10)
C8—C11—H11B	109.5	O1—Cu1—N2	97.50 (10)
H11A—C11—H11B	109.5	O2—Cu1—N2	95.87 (9)
C8—C11—H11C	109.5	N1—Cu1—N2	107.40 (9)
H11A—C11—H11C	109.5	N4—Cu1—N2	84.45 (9)
H11B—C11—H11C	109.5	C7—N1—C8	121.9 (3)
N2—C12—C13	111.0 (3)	C7—N1—Cu1	129.1 (2)
N2—C12—C16	120.2 (3)	C8—N1—Cu1	109.07 (18)
C13—C12—C16	128.8 (3)	C12—N2—N3	104.9 (2)
C14—C13—C12	106.7 (3)	C12—N2—Cu1	134.91 (19)
C14—C13—H13	126.6	N3—N2—Cu1	118.30 (18)
C12—C13—H13	126.6	C14—N3—N2	111.7 (3)
N3—C14—C13	105.7 (3)	C14—N3—C17	130.7 (3)
N3—C14—C15	123.0 (3)	N2—N3—C17	117.5 (2)
C13—C14—C15	131.2 (3)	C20—N4—N5	105.7 (2)
C14—C15—H15A	109.5	C20—N4—Cu1	131.3 (2)
C14—C15—H15B	109.5	N5—N4—Cu1	122.41 (18)
H15A—C15—H15B	109.5	C18—N5—N4	111.2 (2)
C14—C15—H15C	109.5	C18—N5—C17	128.8 (3)
H15A—C15—H15C	109.5	N4—N5—C17	120.0 (2)
H15B—C15—H15C	109.5	C1—O1—Cu1	126.14 (19)
C12—C16—H16A	109.5	C9—O2—Cu1	114.6 (2)
C12—C16—H16B	109.5		

## References

[bb1] Baul, T. S. B., Masharing, C., Ruisi, G., Jirásko, R., Holčapek, M., Voc, D., Wolstenholme, D. & Linden, A. (2007). *J. Organomet. Chem.* **692**, 4849–4862.

[bb2] Bruker (2000). *SMART* and *SAINT* Bruker Axs Inc., Madison, Wisconsin, USA.

[bb3] Casella, L. & Guillotti, M. (1983). *Inorg. Chem.* **22**, 2259–2266.

[bb4] Ganguly, R., Sreenivasulu, B. & Vittal, J. J. (2008). *Coord. Chem. Rev.* **252**, 1027–1050.

[bb5] Parekh, H. M., Mehta, S. R. & Patel, M. N. (2006). *Russ. J. Inorg. Chem.* **35**, 67–72.

[bb6] Plesch, G., Friebel, C., Warda, S. A., Sivý, J. & Švajlenová, O. (1997). *Transition Met. Chem.* **22**, 433–440.

[bb7] Sheldrick, G. M. (1996). *SADABS* University of Göttingen, Germany.

[bb8] Sheldrick, G. M. (2008). *Acta Cryst.* A**64**, 112–122. 10.1107/S0108767307043930 18156677

[bb9] Usman, A., Fun, H.-K., Basu Baul, T. S. & Paul, P. C. (2003). *Acta Cryst.* E**59**, m438–m440.

[bb10] Vigato, P. A. & Tamburini, S. (2004). *Coord. Chem. Rev.* **248**, 1717–2128.

